# *Novel Mutations* in the Transcriptional Activator Domain of the Human TBX20 in Patients with Atrial Septal Defect

**DOI:** 10.1155/2015/718786

**Published:** 2015-03-05

**Authors:** Irma Eloisa Monroy-Muñoz, Nonanzit Pérez-Hernández, José Manuel Rodríguez-Pérez, José Esteban Muñoz-Medina, Javier Angeles-Martínez, José J. García-Trejo, Edgar Morales-Ríos, Felipe Massó, Juan Pablo Sandoval-Jones, Jorge Cervantes-Salazar, José Antonio García-Montes, Juan Calderón-Colmenero, Gilberto Vargas-Alarcón

**Affiliations:** ^1^Department of Molecular Biology, Instituto Nacional de Cardiología Ignacio Chávez, 14080 Mexico City, Mexico; ^2^Central Laboratory of Epidemiology, Instituto Mexicano del Seguro Social, 02900 Mexico City, Mexico; ^3^Department of Biology, Chemistry Faculty, Universidad Nacional Autónoma de México, 04510 Mexico City, Mexico; ^4^Department of Physiology, Instituto Nacional de Cardiología Ignacio Chávez, 14080 Mexico City, Mexico; ^5^Department of Pediatric Cardiology, Instituto Nacional de Cardiología Ignacio Chávez, 14080 Mexico City, Mexico; ^6^Department of Congenital Heart Disease Surgery, Department of Pediatric Cardiology, Instituto Nacional de Cardiología Ignacio Chávez, 14080 Mexico City, Mexico; ^7^Department of Interventional Cardiology, Instituto Nacional de Cardiología Ignacio Chávez, 14080 Mexico City, Mexico

## Abstract

*Background*. The relevance of *TBX20* gene in heart development has been demonstrated in many animal models, but there are few works that try to elucidate the effect of *TBX20* mutations in human congenital heart diseases. In these studies, all missense mutations associated with atrial septal defect (ASD) were found in the DNA-binding T-box domain, none in the transcriptional activator domain. *Methods*. We search for *TBX20* mutations in a group of patients with ASD or ventricular septal defect (VSD) using the High Resolution Melting (HRM) method and DNA sequencing. *Results*. We report three missense mutations (Y309D, T370O, and M395R) within the transcriptional activator domain of human TBX20 that were associated with ASD. *Conclusions*. This is the first association of TBX20 transcriptional activator domain missense mutations with ASD. These findings could have implications for diagnosis, genetic screening, and patient follow-up.

## 1. Introduction

Congenital heart defects (CHD) are the most common developmental defects in humans [1], affecting 6–8 out of 1,000 newborns [2–5]. In Mexico CHD have an estimated prevalence of 1% (10 out of 1,000 newborns) [6]. CHD are a group of multifactorial complex diseases with environmental and genetic factors playing important roles [7]. Mutations in genes, such as in the transcription factors genes* NKX2.5*,* TBX5,* and* GATA4*, have been correlated to the pathogenesis of CHD [8]. Although the research of the genes involved in cardiac developmental pathways is growing we are not able to completely define how mutations in these genes cause CHD.

The heart is formed in early stages of embryonic development. This process requires the action of several transcription factors that regulate through activation or repression of key genes in a specific temporary/spatial manner [7]. An important group of transcription factors involved in heart development is the T-box family.* T-box* genes mutations in humans are associated with CHD [9–11]. The action of TBX20, a member of the TBX1 subfamily of T-box proteins, is necessary in early stages of heart development, by coordinating cardiomyocyte proliferation and regional specification and formation of cardiac chambers and valves [12]. Direct downstream target genes of TBX20 in the primitive myocardium include* TBX2* and* N-myc1* that function to regulate cardiomyocyte proliferation [13].

In adult mice, heterozygous loss of* TBX20* leads to dilated cardiomyopathy [10] and the conditional homozygous loss of* TBX20* in cardiomyocytes results in severe cardiomyopathy with associated arrhythmias and death [14]. In 2013, studies in mice showed that* TBX20* mutations resulted in failure of heart looping, developmental arrest, and lack of chamber differentiation [15].

Mutations in human* TBX20* that result in gain or loss of protein function are associated with a wide array of cardiac malformations, including septal defects, defects in valvulogenesis, and cardiomyopathy [1, 11, 16]. TBX20 carries strong transcriptional activation and repression domains, and it physically or genetically interacts with other cardiac developmental transcription factors, including NKX2-5, GATA4, GATA5, and TBX5. There have been several associations of TBX20 missense mutations with ASD. All mutations were found in the DNA-binding T-box domain, none in the transcriptional activator domain. We therefore screened 38 CHD-affected subjects for* TBX20 *mutations and found three missense mutations that lay within exons encoding the transcriptional activator domain. We also found ten nonsense mutations and one nonreported SNP. These last findings could contribute to the risk of CHD.

## 2. Material and Methods

### 2.1. Study Population

Thirty-eight patients with atrial septal defect (ASD) or ventricular septal defect (VSD) attended the Department of Pediatric Cardiology at the Instituto Nacional de Cardiología ‘‘Ignacio Chávez” (INCICH). All septal heart defects were corrected with an Amplatzer septal occluder device by transcatheterization. Patients who were diagnosed with syndromatic heart defects were excluded from the study. They were unrelated individuals recruited without reference to family history during 2011-2012. Subjects included as control group underwent echocardiography to exclude CHD. Informed written consent was obtained from all recruited patients and controls. On behalf of the children enrolled in our study we obtained written informed consent from their guardians. The study complies with the Declaration of Helsinki and was approved by the Ethics Committee of the INCICH.

### 2.2. Mutational Analysis

Genomic DNA was isolated from peripheral blood leukocytes using standard techniques. The High Resolution Melting (HRM) method was used to detect mutations and SNPs in* TBX20* gene. The HRM primers were designed using Primer Select program (DNASTAR), which also evaluates that primers sequence do not form secondary structures during PCR that can increase the complexity of melting profile interpretation. The primers specificity was tested through PrimerBlast platform (NCBI). Primers were designed to amplify complete exonic sequences and small flanking intronic sequences (Table 1). Reactions were performed with a total volume of 20 uL (5 uL of Mili-Q water, 1.5 uL of each primer at 20 pmol/uL, 10 uL of SSoFast Eva Green Master Mix (Biorad), and 2 uL of DNA at 150 ng/uL). The amplification parameters were 95°C for 4 minutes, 30 cycles of 94°C for 30 seconds annealing temperature for 30 seconds, and 72°C for 30 seconds, followed by a final extension step of 72°C for 5 minutes. For melting curve analysis, the parameters were 95°C for 30 seconds and 75°C for 30 seconds. Data were collected over a temperature range of 75–95°C in 0.1°C increments every 10 seconds.

### 2.3. DNA Sequencing

After purification with Exosap, HRM products were sequenced using Big Dye Terminator v1.1 and v3.1 kits (Applied Biosystems) and ABI PRISM 3130 DNA Analyzer. Resultant sequences were analyzed with BioEdit software and nBlast platform (NCBI). Nucleotide sequences were translated using the translate tool in ExPASy Bioinformatics Resource Portal. Whenever a sequence variant was found, the sample was sequenced again from the opposite direction to confirm the nucleotide change. The effect of the mutations was evaluated* in silico*, using PolyPhen software v2.0.23 http://genetics.bwh.harvard.edu/pph2/. PolyPhen is a tool that predicts possible impact of an amino acid substitution on the structure and function of a human protein using straightforward physical and comparative considerations.

### 2.4. Molecular Modeling

The homology model of TBX20 was produced using the protein structure homology modeling server SWISS-MODEL and based on the structure of the T-box domain of human TBX3 (PDB 1h6f). Graphics were generated using PyMol.

## 3. Results

### 3.1. Missense Mutations

**Figure 1:**
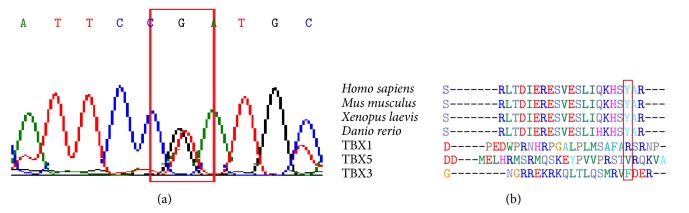
(a) The relevant sequence electropherogram of* tbx20* (NM_001077653.2) in Exon 7 of the subject. (b) The affected amino acid (Y309D) lies in a highly conserved C-terminal region of the transcriptional activator domain of TBX20. Affected region of TBX20 homologues and human TBX paralogues are shown. The variants are highlighted with a red rectangle.

One heterozygous transition T→G at position c.925 (NM_001077653.2) was detected in a 71-year-old female subject with ASD (Figure 1(a) and Table 2). The variant resulted in a missense mutation, a shift from tyrosine to aspartate (Y309D). It was found in 1 of the 41 cases and was not seen in the control group. This mutation was located in the transcriptional activator domain, and it is highly conserved among species, but not among other TBX proteins (Figure 1(b)). It was not possible to generate a homology model because the available templates only included 279 amino acid residues. Using program PMut, which predicts whether an amino acid substitution affects protein function, Y309D was defined as pathological mutation. With the* in silico* studies based on PolyPhen 2 (Polymorphism Phenotyping v2), we confirmed that Y309D is probably damaging.

**Figure 2:**
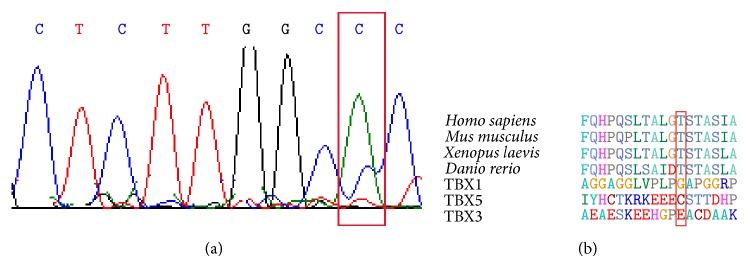
(a) The relevant sequence electropherogram of A→C at position c.1108 (NM_001077653.2) in the subject (Exon 8). (b) The affected amino acid (T370P) lies in a highly conserved C-terminal region of the transcriptional activator domain of TBX20 among species. The variants are highlighted with a red rectangle.

**Figure 3:**
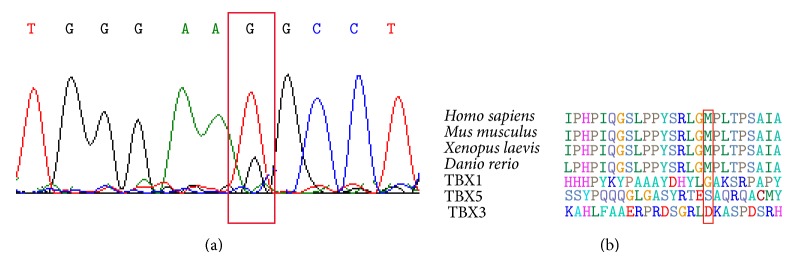
(a) T→G at position c.1184 (NM_001077653.2) in the subject (Exon 8). (b) The affected amino acid (M395R) lies in a highly conserved C-terminal region of the transcriptional activator domain of TBX20 among species. The variants are highlighted with a red rectangle.

Two heterozygous transitions were detected only in a seven-year-old female subject with ASD. The first one was a change from A→C at position c.1108 (NM_001077653.2) (Figure 2(a) and Table 2), a shift from threonine to proline in the TBX20 protein (NP_001071121.1:p.T370P) (Figure 2(b) and Table 2). The second one was a change from T→G at position c.1184 (NM_001077653.2) (Figure 3(a) and Table 2). This variant resulted in a missense change TBX20 M395R (NP_001071121.1) (Figure 3(b) and Table 2). Both mutations were classified by PolyPhen software as benign (Table 2). These two mutations were located in the transcriptional activator domain and are highly conserved across species, but not among other TBX proteins (Figures 2(b) and 3(b)).

### 3.2. Synonymous and Noncoding Sequence Variants

Heterozygous transition C→T at position c.−517 (NM_001077653.2) was detected in a 2-year-old female subject with VSD. This variation was located in the 5'UTR region, and it was found only in this subject (Table 3).

Two synonymous variants were found in the group of patients and were absent in the control group. The first one was heterozygous transversion A→C at position c.657 (NM_001077653.2) found in three patients. The second one was heterozygous transition C→T at position c.1189 (NM_001077653.2) (Table 3) found in two patients.

Three changes that modified polyadenylation sites were found in the patients group. Three heterozygous transversions: A→T at position c.1356 (NM_001077653.2:c.^*^12A>T), T→A at position c.1357 (NM_001077653.2:c.^*^13T>A), and T→A at position c.1392 (NM_001077653.2:c.^*^48T>A) (Table 3).

Homozygous duplication of a timine was found in a twelve-year-old female patient with ASD, at position c.546−1223dup (NM_001077653.2) (Table 3).

Two heterozygous transversions were found in a 71-year-old female subject with ASD. The first one was at position c.1003+99C>T (NM_001077653.2) and the second one was at position c.1003+129T>C (NM_001077653.2) (Table 3).

### 3.3. Single Nucleotide Polymorphisms (SNPs)

We also detected twelve SNPs both in CHD patients and controls. Eleven of them have been listed in database of SNP lists. Only one, the one found in intron 4, has not been reported previously (Table 4).

**Figure 4:**
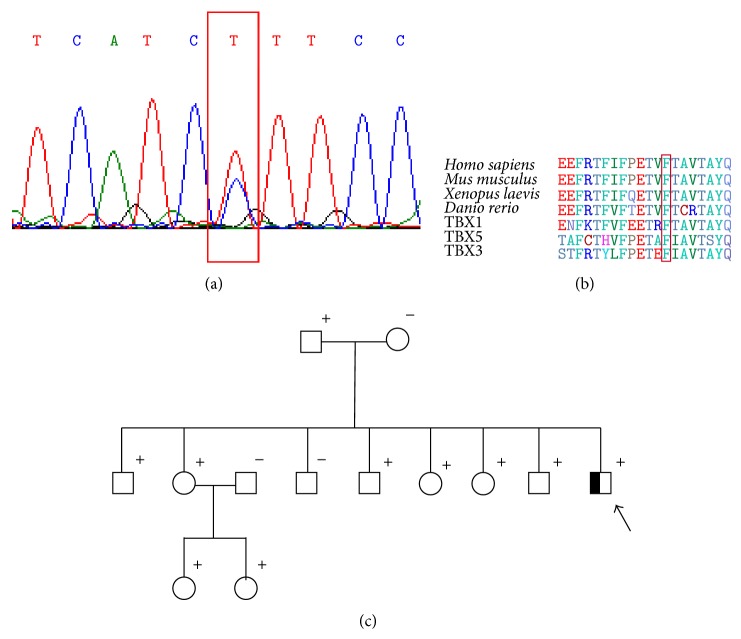
(a) C→T at position c.766 (NM_001077653.2) in the subject (Exon 5). The affected amino acid (F256L) is a highly conserved residue outside the DNA-binding T-box domain region of the transcriptional activator domain of TBX20 among species. The variants are highlighted with a red rectangle. (c) Family pedigree of mutation carriers. The subject is marked with an arrow. All subjects which were genotyped for TBX20-F256L are indicated with + (carrier) or − (noncarrier).

**Figure 5:**
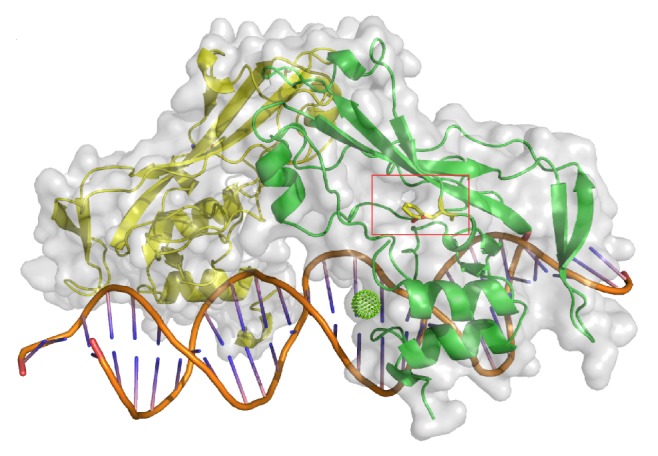
The homology model of TBX20, based on the structure of human TBX3, suggests that the presence of the F256L SNP diminishes in the hydrophobic surface of the protein. The variant is highlighted with a red rectangle.

One heterozygous transition C→T at position c.766 (NM_001077653.2) was detected in an 11-year-old male subject with ASD (Figure 4(a) and Table 4). The variant resulted in a shift from phenylalanine to leucine in TBX20 protein (F256L) (Figure 4(b) and Table 4). This variant was already described as rs3999941 SNP. The c.766T>C was present in 9 members of his family, without any diagnosis of CHD (Figure 4(c)). As shown in Figure 5 the affected residue lies outside the DNA-binding T-box domain generating hydrophobic surface decrease of the protein (Figure 5). Analysis with PolyPhen 2 suggests that most of these missense variants are probably damaging.

## 4. Discussion

Most missense mutations found in TBX20 are located in the DNA-binding T-box domain [9]. The only two mutations described in the transcriptional activation domain were reported in patients with dilated cardiomyopathy [17]. We identified three unique missense* TBX20* mutations in the transcriptional activation domain in two ASD subjects (Table 2). So this is the first report of* TBX20* mutations in the transcriptional activation domain in ASD patients. Neither mutation was found in our control group. These changes occurred in highly conserved amino acid among species.* In silico* analysis showed a pathological effect only of the Y309D mutation (NM_001077653.2:c.927G>T) (Table 2). This could be the result of its proximity to the DNA-binding T-box domain, so the amino acid change could have a higher effect in the protein function. The remaining two mutations (T370P and M395R) were classified by PolyPhen software as benign and are conserved across species. The three mutations could have an effect over TBX20 activity as modifiers of the affinity of the T-box domain to the T-site, because crystallographic and* in vitro *binding studies revealed that some T proteins can bind to DNA as dimers and that its T-domain forms a new type of specific DNA contact, in which a carboxyterminal helix is deeply embedded into an enlarged minor groove without bending the DNA [18, 19]. Also, transcriptional activator (C-terminal region) domains of transcriptional factors are necessary to establish an interaction with the basal transcriptional machinery, and a change in this domain could affect its activity, as it was found in 2007 by Farin and colleagues. They performed a molecular analysis of TBX15 and TBX18 proteins, and they found that N-terminal and C-terminal regions participate in protein-DNA complex formation [20].

At the present time there are no outstanding findings that demonstrate the association of* TBX20* mutations with VSD. There is only one report of a missense change TBX20 I152M (456C→G) in a VSD subject [16], so in order to evaluate if there was an association in our population, we decided to include patients with VSD in the mutational analysis. Our findings showed the presence of only one noncoding change in the 5'UTR region in a subject with VSD, which was not found in the control group. As we do not know the effect of this mutation, the association between* TBX20* mutations and VSD in our population remains unclear.

The detection of synonymous, noncoding mutations, and SNPs in addition to missense mutations should be considered regarding ASD severity [21]. Some SNPs resulted in an amino acid change (c.766T>C, c.925T>A, and c.1331C>T) and one of them is a splicing donor variant (c.813+1G>A). F256L (c.766T>C) lies in a residue, which was considered as part of the DNA-binding domain [1], but the homology model of TBX20, based on the structure of human TBX3, suggests that it is located outside the T-box domain (Figure 5). The F256L seems to reduce the hydrophobic surface of the protein. Site-directed mutagenesis analysis where bulky hydrophobic residues like leucine were replaced by smaller residues as alanine demonstrated that these replacements destabilize the protein not only because there is the reduction in hydrophobic stabilization of alanine to leucine, but also because there is an energetic cost associated with the creation of a cavity in the folded protein. When a large cavity is created, the replacement is more destabilizing [22]. Despite not being part of the T-box domain, the change from phenylalanine to leucine generated by c.766T>C could affect TBX20 protein, by destabilization.

Due to its complexity it is very difficult to associate a CHD with a single mutation in a key gene in heart development, such as* TBX20*. Our findings provide the first insight into missense mutations of TBX20 transcriptional activator domain associated with ASD. Functional studies of the new variants of TBX20 identified in the present study should be subject of further investigation.

## Figures and Tables

**Table 1:** Exons 1 to 8 primers sequences.

Exon	Forward	Reverse
1a	5′-GATCGCCGCCGCCAGCAAAT-3′	5′-AGGAGAGGGCCCACCGAGCACTAC-3′
1b	5′-GTAGTGCTCGGTGGGCCCTCTCCT-3′	5′-GCGTTGGCCCGAGAGGAGAGTTGG-3′
1c	5′-CCAACTCTCCTCTCGGGCCAACGC-3′	5′-GCACATTCACAGCATTCAACAGAC-3′
2	5′-CATTTGGTTATGCTGTTCTTTCC-3′ [16]	5′-CTACCCAGGGAGTGTCCTG-3′ [16]
3	5′-GTTTGTGGACCGGATAGAGA-3′	5′-CAGGCTTGGAATGCTCTCTT-3′
4	5′-ACTTATATATGGTTTATGTGTT-3′	5′-GGTCCCCTGAAGAACACATAAAAT-3′
5	5′-CACTGTAATTTGGCCTGTTTAGC-3′ [16]	5′-AATATAAGAACCTCCTAAATCCTTCTC-3′ [16]
6	5′-TTCCACCCTTCTCAGGACAC-3′ [16]	5′-AGGCCTGCCTGATGTCTCT-3′ [16]
7	5′-AGTGGTTGCTTTTTGGCTGAGA-3′	5′-TCAAAGGCAAAATAATGAAATCTG-3′
8	5′-CAGTGTTTCCAGTCTAATGAGTGT-3′	5′-AGTCTGGCTCTCCTCTTTGAT-3′

**Table 2:** Missense mutations detected in this cohort of CHD patients.

Nucleotide change	Amino acid change	Exon	Number of Patients	Cardiac defects	PolyPhen 2
c.925T>G	Y309D	7	1	ASD	D
c.1108A>C	T370P	8	1	ASD	B
c.1184T>G	M395R	8	1	ASD	B

ASD: atrial septal defect; PMut: a program predicting whether an amino acid substitution affects protein function; B: benign; D: damaging.

**Table 3:** Synonymous and noncoding sequence variants in this cohort of CHD patients.

Nucleotide change	Location	Amino acid change	Number of patients	Cardiac defect
c.−517C>T	5′UTR	Non	1	VSD
c.657A>C	Exon 5	I219I	3	ASD
c.1189C>T	Exon 8	L397L	2	ASD
c.1356A>T (NM_001077653.2: c.^*^12A>T)	3′UTR	Non	2	ASD
c.1357T>A (NM_001077653.2: c.^*^13T>A)	3′UTR	Non	2	ASD
c.1392T>A (NM_001077653.2: c.^*^48T>A).	3′UTR	Non	1	ASD
c.546−1223dup	Intron 3	Non	1	ASD
c.1003+99C>T	Intron 7	Non	1	ASD
c.1003+129T>C	Intron 7	Non	1	ASD

VSD: ventricular septal defect; ASD: atrial septal defect; UTR: untranslated region.

**Table 4:** SNPs detected in this cohort of CHD patients.

Nucleotide change	Location	Effect	SNP name	Cardiac defect
c.655−18C>T	Intron 4	Non	Novel	ASD
c.−186T>C	5′UTR	Non	rs73099190	ASD
c.766T>C	Exon 5	Missense variant (F256L)	rs3999941	ASD
c.813+1G>A	Intron 6	Splicing donor variant	rs3999940	ASD
c.890+128C>T	Intron 7	Intron variant	rs2109090	ASD
c.891−55G>C	Intron 7	Intron variant	rs111666016	ASD
c.925T>A	Exon 7	Missense variant (Y309N)	rs111862418	ASD
c.891−30C>G	Intron 6	Intron variant	rs113178075	ASD
c.1164A>G	Exon 8	Synonymous variant (P388P)	rs2723759	ASD
c.1194A>C	Exon 8	Synonymous variant (T398T)	rs2532122	ASD
c.1331C>T	Exon 8	Missense variant (T444M)	rs201217462	ASD
c.655−44G>A	Intron 4	Intron variant	rs2072434	ASD

ASD: atrial septal defect; UTR: untranslated region.
